# Prevention of Contrast-Induced Acute Kidney Injury: Is Simple Oral Hydration Similar To Intravenous? A Systematic Review of the Evidence

**DOI:** 10.1371/journal.pone.0060009

**Published:** 2013-03-26

**Authors:** Swapnil Hiremath, Ayub Akbari, Wael Shabana, Dean A. Fergusson, Greg A. Knoll

**Affiliations:** 1 Division of Nephrology, Faculty of Medicine, University of Ottawa, Ottawa, Ontario, Canada; 2 Clinical Epidemiology Program, Ottawa Hospital Research Institute, Ottawa, Ontario, Canada; 3 Department of Medical Imaging, Faculty of Medicine, University of Ottawa, Ottawa, Ontario, Canada; University of Washington School of Medicine, United States of America

## Abstract

**Background:**

Pre-procedural intravenous fluid administration is an effective prophylaxis measure for contrast-induced acute kidney injury. For logistical ease, the oral route is an alternative to the intravenous. The objective of this study was to compare the efficacy of the oral to the intravenous route in prevention of contrast-induced acute kidney injury.

**Study Design:**

A systematic review and meta-analysis of randomised trials with a stratified analysis and metaregression. Databases included MEDLINE (1950 to November 23 2011), EMBASE (1947 to week 47 2011), Cochrane CENTRAL (3^rd^ quarter 2011). Two reviewers identified relevant trials and abstracted data.

**Settings and Population:**

Trials including patients undergoing a contrast enhanced procedure.

**Selection Criteria:**

Randomised controlled trial; adult (>18 years) population; comparison of oral versus intravenous volume expansion.

**Intervention:**

Oral route of volume expansion compared to the intravenous route.

**Outcomes:**

Any measure of acute kidney injury, need for renal replacement therapy, hospitalization and death.

**Results:**

Six trials including 513 patients met inclusion criteria. The summary odds ratio was 1.19 (95% CI 0.46, 3.10, p = 0.73) suggesting no difference between the two routes of volume expansion. There was significant heterogeneity (Cochran’s Q = 11.65, p = 0.04; I^2^ = 57). In the stratified analysis, inclusion of the five studies with a prespecified oral volume expansion protocol resulted in a shift towards oral volume expansion (OR 0.75, 95% CI 0.37, 1.50, p = 0.42) and also resolved the heterogeneity (Q = 3.19, P = 0.53; I^2^ = 0).

**Limitations:**

Small number of studies identified; lack of hard clinical outcomes.

**Conclusion:**

The oral route may be as effective as the intravenous route for volume expansion for contrast-induced acute kidney injury prevention. Adequately powered trials with hard endpoints should be done given the potential advantages of oral (e.g. reduced patient burden and cost) over intravenous volume expansion.

## Introduction

Contrast-enhanced radiological procedures are invaluable from a diagnostic and therapeutic perspective, with more than 80 million doses of iodinated contrast administered annually worldwide [1]. However, iodinated contrast can cause acute kidney injury and is the leading cause of iatrogenic, and hence preventable, acute kidney injury [2], [3]. Severe acute kidney injury requires dialysis and results in increased morbidity and mortality, prolonged hospitalization and overall increased healthcare resource utilization [3], [4], [5], [6]. Of the various prophylactic measures tested in clinical trials, intravenous near-isotonic fluid administration has stood out as the having the most consistent therapeutic benefit [7], [8], [9], [10], [11], [12], [13].

Adequate volume expansion before and after contrast administration improves renal blood flow and thus attenuates the negative hemodynamic conditions which lead to the development of contrast-induced acute kidney injury (CI-AKI) [14], [15]. Randomized controlled trials have demonstrated the benefit of near-isotonic fluids over hypotonic fluids and the lack of added protection with a bicarb-based solution over saline [15], [16], [17]. However, administration of intravenous solutions requires nursing time, a stay in the hospital daycare for outpatient procedures and increased healthcare resource utilization [18], [19], [20]. Contrast-enhanced imaging does not always occur in the hospital setting, since computed tomography (CT) is often performed in centers located outside the hospital [21]. Not surprisingly, despite recommendations from guidelines, only 45% of patients undergoing coronary angiogram received intravenous fluids in a prospective observational study [22].

The oral route of volume expansion is an alternative to intravenous fluids and has the added advantage of logistical ease and lower healthcare resource utilization [18]. However, efficacy of the oral route of volume expansion in CI-AKI prevention has not been established [11], [12]. Therefore, we conducted a systematic review of randomized controlled trials comparing oral versus intravenous route of volume expansion for prevention of CI-AKI amongst patients who were administered contrast media.

## Methods

### Study Objectives and Design

The primary aim of the systematic review was to determine if the incidence of CI-AKI differed between patients who received volume expansion administered orally versus by the intravenous route. The primary outcome of interest was development of CI-AKI; as the definition of CI-AKI varied, we used the definition employed by the investigators in each trial. Secondary outcomes included death, hospitalization and need for renal replacement therapy.

### Data Sources and Searches

We conducted a systematic literature search of MEDLINE (1950 to November 23 2011), EMBASE (1947 to week 47 2011) and Cochrane CENTRAL (until 3^rd^ quarter 2011) for randomized controlled trials of volume expansion to prevent CI-AKI with the assistance of a librarian (see details of the search strategy for the MEDLINE search in supplementary information, Table S1). In addition, we searched the reference lists of all identified relevant publications, reviews and prior meta-analyses of CI-AKI. We considered articles published in any language.

### Study Selection

Two reviewers (SH and AA) identified articles for further review by performing an initial screen of identified abstracts or titles. Articles were considered for inclusion in the systematic review if they were randomized trials of pre-procedural volume expansion comparing the oral versus the intravenous route and reporting CI-AKI as an outcome in the adult population (≥18 years). Articles identified by either reviewer were retained. The full text for the articles was then obtained to perform a second screening. Differences regarding study inclusion were resolved by discussion and input of a third investigator if needed.

### Data Extraction and Quality Assessment

We extracted pre-specified data elements from each trial including: study design, volume expansion protocol, patient characteristics and setting (e.g. cardiac catheterization), sample size, primary endpoint, secondary outcome measures, contrast type and contrast volume. If there were more than two treatment arms, the groups were condensed when possible into one intravenous and one oral group. Study characteristics and measures of quality were also identified a priori for inclusion in stratified analysis and meta-regression. Quality measures included the Jadad score [23] and allocation concealment. Study characteristics included baseline kidney function, average age, proportion of diabetic patients included, and average contrast volume.

### Data Synthesis and Analysis

We calculated the Peto odds ratio for development of CI-AKI given the low event rates [24]. The average effects for the outcomes and 95% confidence intervals were obtained using a random effects model as described by DerSimonian and Laird [25]. We chose the random effects method because of its conservative summary estimate. To assess heterogeneity, we used the Cochran’s Q statistic test (P-value <0.1 considered significant) and the I^2^ statistic. Sensitivity analyses were performed to assess the effects of selected measures of study quality and clinical factors. Stratified analyses and meta-regression were applied to determine if prespecified covariates (mean age, proportion of diabetics, baseline kidney function, and mean contrast dose) might explain heterogeneity of results among the studies. A funnel plot was used to assess for the presence of publication and other reporting biases by plotting the standard error against the log odds ratio and the Egger’s statistic was used to test for asymmetry [26].

The two-tailed P-value threshold for statistical significance was set at 0.05 for effect sizes. Analyses were conducted using Comprehensive Meta-analysis software (version 2.2.046, Biostat Inc, Englewood, NJ). The study was performed in accordance to the recommendations set forth by the Preferred Reporting Items for Systematic Reviews and Meta-Analyses (PRISMA) workgroup (see Table S2 for PRISMA checklist) [27], [28].

## Results

### Literature Search

**Figure 1:**
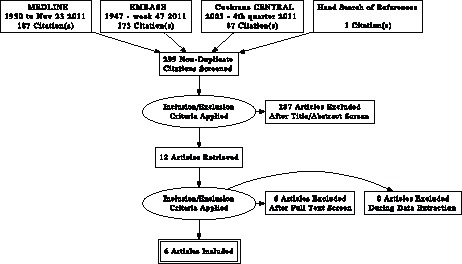
PRISMA Flow diagram.

The search yielded 299 non-duplicate citations, comprising 187 articles from MEDLINE, 173 from EMBASE and 87 from Cochrane CENTRAL and one study identified from the references of a review article. The majority of the articles retrieved in the search were not relevant because they were controlled trials of other interventions (e.g. type of contrast media, n-acetylcysteine etc) or were opinion-based review articles. Of the 12 systematic reviews and meta-analyses identified in this search, none addressed the question posed in this study (i.e. IV versus oral volume expansion). Of the 299 articles, 12 were potentially eligible and reviewed as full-text articles. Six studies were excluded for the following reasons: no clinical outcomes reported (n = 1); comparison of volume expansion with normal versus half normal saline (n = 1); comparison of pre-procedural versus no pre-procedural volume expansion (n = 1); comparison of post-procedural volume expansion strategies (n = 1); comparison of volume expansion and N-acetylcysteine versus no volume expansion (n = 1) and a non-randomized study (n = 1). A total of six trials involving 513 patients were included in the primary analysis (Figure 1) [18], [29], [30], [31], [32], [33]. There was perfect agreement between the two reviewers on article selection.

### Study Characteristics

The clinical and methodological characteristics of the six studies included are presented in Tables 1 and 2. In general, the studies were small, with a median sample size of 85 (range 36 to 153). Four studies were in the setting of cardiac catheterization [18], [31], [32], [33], one was in patients undergoing angiography for peripheral vascular disease [30] and another study included patients undergoing a mix of procedures including contrast enhanced CT scans [29]. The studies included patients with mild to moderate chronic kidney disease at baseline (range of baseline serum creatinine, 1.14 to 2.35 mg/dl). The average amount of contrast volume administered also varied from 101 ml to 201 ml. In five trials, the definition of CI-AKI was increase in creatinine from baseline of at least 25% or 44 µmol/L at 48 or 72 hours, which is the most common CI-AKI definition [34]. In one trial, the definition was an increase in creatinine of 0.3 mg/dL [35], which would be equivalent to Stage 1 AKI as per the Acute Kidney Injury Network (AKIN) criteria [35].

**Table 1:** Tabl**e 1.** Clinical Setting and Hydration Protocols of Included Trials.

Study (year)	Contrast Procedure	Contrast Type	Intravenous Regimen	Oral Regimen	Study Definition of CI-AKI
Taylor (1998)	cardiac catheterization	Ionic (74%) Nonionic (24%)	0.45% saline 75 mL/hourfor 12 hours before and12 hours after	1000 mL water over 10 hours beforeand IV 0.45% saline after for 6 hours	Increase in creatinine from baseline of at least 26.4 µmol/L (0.3 mg/dl) within 48 hours
Trivedi (2003)	nonemergency cardiac catheterization	Low-osmolality, Ionic	1 mL/kg/hour of isotonic saline for 12 hours before	Allowed unrestricted oral fluids	Increase in creatinine from baseline of at least 44.2 µmol/L (0.5 mg/dl) within 48 hours
Dussol (2006)	various radiological procedures	Low-osmolality, Non-ionic	15 mL/kg isotonic saline over 6 hours before	1 g/10 kg body weight NaCl for 2 days before	Increase in creatinine from baseline of at least 44 µmol/L (0.5 mg/dl) within 48 hours
Lawlor (2007)	elective, outpatient angiography	NR*	1 mL/kg/hour isotonic saline 12 hours beforeand 12 hours after	1000 mL water over 12 hours beforeand IV saline 1 mL/kg/hour for12 hours post	Increase in creatinine from baseline of at least 25% or 44 µmol/L (0.5 mg/dl) at 48 hours
Cho(2010)	elective coronary angiogram	Low-osmolality, Non-ionic (isoversol)	3 mL/kg bolus of isotonicsaline orsodium bicarbonate1 hour before and1 mL/kg/hour for6 hours post	500 mL water 4 hours before, stopped2 hours before; 3.9 g oral NaHCO_3_20 minutes before; 600 mL ofwater post procedure with 1.95 gNaHCO_3_ at 2 and 4 hours or500 mL water 4 hours before,stopped 2 hours before and600 mL of water post procedure	Increase in creatinine from baseline of at least 25% or 44 µmol/L (0.5 mg/dl) at 72 hours
Wrobel (2010)	percutaneous coronaryintervention	Low-osmolality, Non-ionic (isoversol)	Isotonic saline,1 mL/kg/hour for6 hours before and12 hours after	Oral mineral water 1 mL/kg/hour for6–12 hours before and 12 hours after	Increase in creatinine from baseline of at least 25% or 44 µmol/L (0.5 mg/dl) at 72 hours

* NR, not reported; NaCl, sodium chloride; NaHCO_3_, sodium bicarbonate.

*Note:* Conversion factors for units: serum creatinine in mg/dL to mol/L, ×88.4.

**Table 2:** Population Characteristics of Included Trials.

	**Total N**	**Baseline Serum Creatinine** **(mg/dL)**		**Male Gender (%)**	**Age (years)**	**Diabetes (%)**	**Contrast Volume (mL)**	
		**IV**	**Oral**				**IV**	**Oral**
Taylor (1998)	36	1.74±0.44	1.75±0.35	81	70	39	177±75	172±60
Trivedi (2003)	53	1.14±0.24	1.27±0.37	98	68	19	201±92	187±88
Dussol (2006)	153	2.35±0.95	2.15±0.74	71	64	29	115±57	120±40
Lawlor (2007)	78	1.92	1.89	69	NR*	NR*	160.5	165
Cho (2010)	91	1.40	1.35	50	79	41	129.4	127.5
Wrobel (2010)	102	1.24±0.45	1.17±0.39	NR	66	NR	101.1±37	110.4±65

Values are means ± standard deviation;

* NR, not reported.

*Note:* Conversion factors for units: serum creatinine in mg/dL to mol/L, ×88.4.

The volume expansion protocols varied for each study. For intravenous volume expansion, isotonic saline was the most commonly used solution (n = 4 trials) [18], [29], [30], [32], [33]. One trial used 0.45% saline [31] and another trial used either saline or sodium bicarbonate [18] (these arms were pooled) for intravenous volume expansion. The oral volume expansion arm varied as well. One study allowed unrestricted fluid intake without a prespecified protocol [32]. Two other studies had pre-procedure oral water and post-procedure intravenous volume expansion in the oral arm [30], [31]. Only two studies included sodium intake [18], [29], in the form of either oral sodium chloride or sodium bicarbonate, to accompany the oral volume expansion.

The quality of the trials was overall quite low, mainly due to the lack of blinding. On the Jadad scale, the trial quality ranged from one (2 trials) to three (4 trials). A similar result was seen with the risk of bias tool (Table 3). Lack of blinding created a high risk of bias across all the studies.

**Table 3:** Quality Assessment of Included Trials.

Trial	Random sequence generation	Allocationconcealment	Blinding of participantsand personnel	Blinding of outcome assessment	Incompleteoutcome data	Selective reporting	Other bias	Jadad Scale
Taylor (1998)	+	+	−	?	+	+	+	3
Trivedi (2003)	+	+	−	?	+	+	−	3
Dussol (2006)	+	+	−	?	+	+	+	3
Lawlor (2007)	+	+	−	?	+	+	+	3
Cho (2010)	?	?	−	?	+	+	+	1
Wrobel (2010)	?	?	−	?	+	+	?	1

### Incidence of CI-AKI

**Figure 2:**
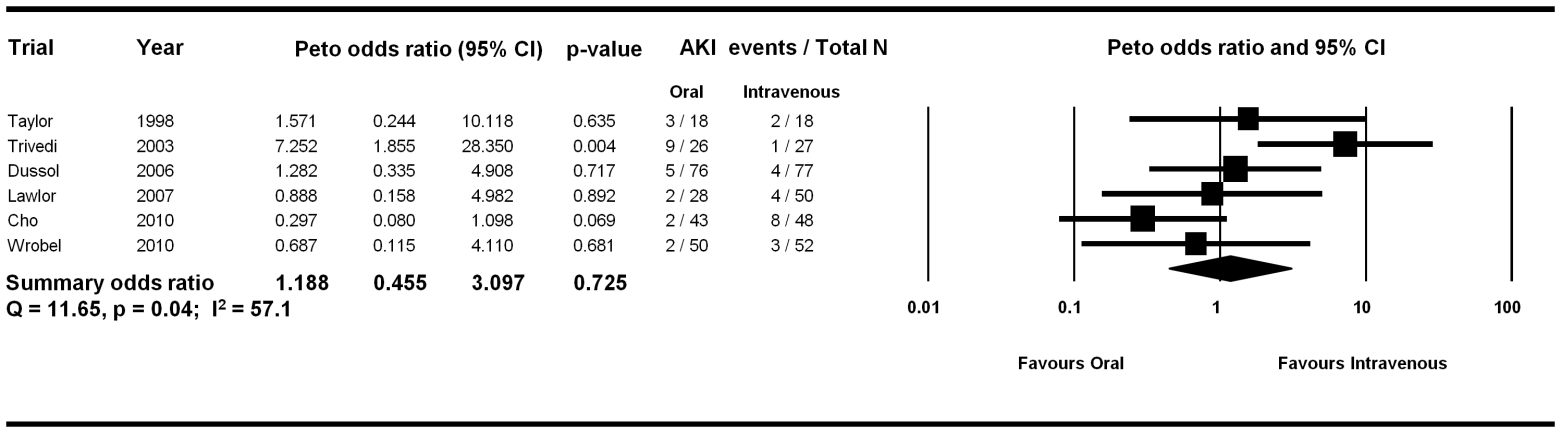
Forest plot of randomized trials meeting inclusion criteria. Size of data markers indicates the weight of the trial. Trials are ordered by year.

**Figure 3:**
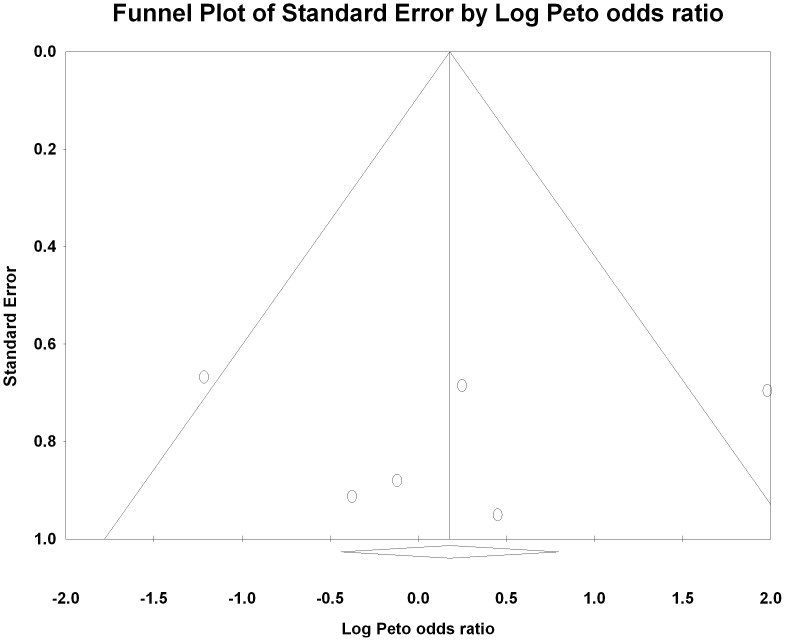
Funnel Plot showing the Peto log odds ratio on the x-axis and the standard error on the y-axis. There is no obvious asymmetry to suggest missing unpublished trials.

Five of the six trials reported no significant difference in the incidence of CI-AKI between the two arms. The only study which reported a significant reduction in CI-AKI with intravenous volume expansion as compared with the oral route was the study which did not have a prespecified protocol for oral volume expansion [32]. The total number of CI-AKI events was small (n = 45 across 6 trials) with a median of eight events per trial (range 5 to 10). The summary Peto odds ratio was 1.19 (95% CI 0.46, 3.10, P = 0.73) suggesting no difference between oral and intravenous volume expansion for CI-AKI prevention (Figure 2). There was no evidence of publication bias by visual examination of the funnel plot (Figure 3) and computing the Egger’s regression intercept (p = 0.34), although there were only a few studies, which were all of a small size.

### Heterogeneity and Stratified Analysis

**Figure 4:**
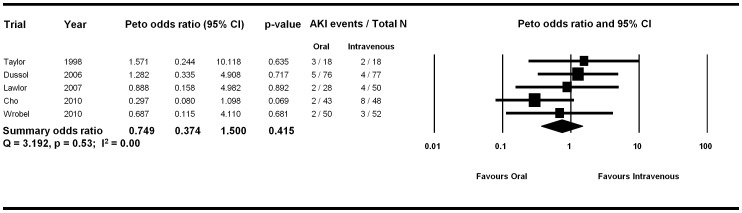
Forest plot including only trials with a prespecified protocol for oral volume expansion showing resolution of heterogeneity.

There was significant heterogeneity amongst the studies (Q = 11.65, p = 0.04; I^2^ = 57%). We carried out stratified analyses based on the oral volume expansion protocols. Firstly, exclusion of the study without a prespecified oral volume expansion protocol resulted in a shift of the summary OR in the direction of a benefit with oral volume expansion (OR 0.75, 95% CI 0.37, 1.50, p = 0.42) and also resolved the heterogeneity (Q = 3.19, p = 0.53; I^2^ = 0%) (Figure 4). Exclusion of the two studies which included post-procedure intravenous volume expansion in the oral arm did not significantly change the effect size (OR 1.19, 95% CI 0.29, 4.83, p = 0.81) or decrease heterogeneity (Q = 11.45, p = 0.01; I^2^ = 73.8). We did stratified analyses based on the use of sodium along with water (n = 2 trials) versus water alone (n = 4 trials). There was a qualitative difference in the summary effect measure that was not statistically significant. The two trials with sodium and water protocols had a pooled OR of 0.61 (95% CI 0.15, 2.57, p = 0.50) favoring oral volume expansion while the four trials with oral water alone protocols had a pooled OR of 1.80 (95% CI 0.57, 5.69, p = 0.32) favoring intravenous volume expansion.

### Metaregression, Sensitivity and Influence Analysis

**Figure 5:**
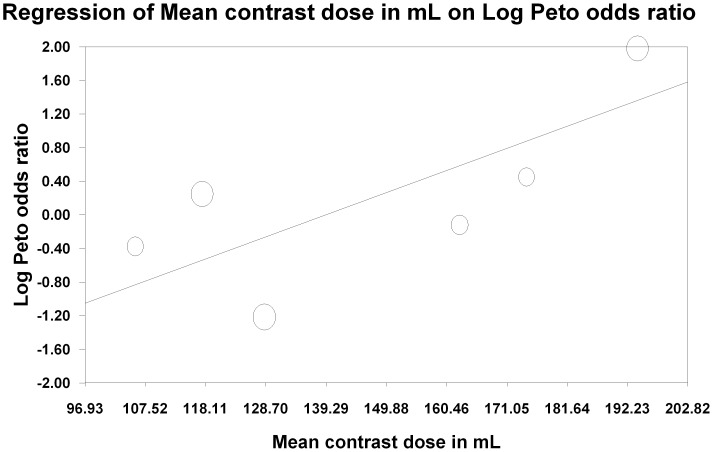
Metaregression of the average total contrast dose administered on the X-axis against the log Peto odds ratio on the Y-axis. This shows that there is a significant trend towards lower CI-AKI with intravenous expansion compared to oral expansion as the contrast dose increases.

**Figure 6:**
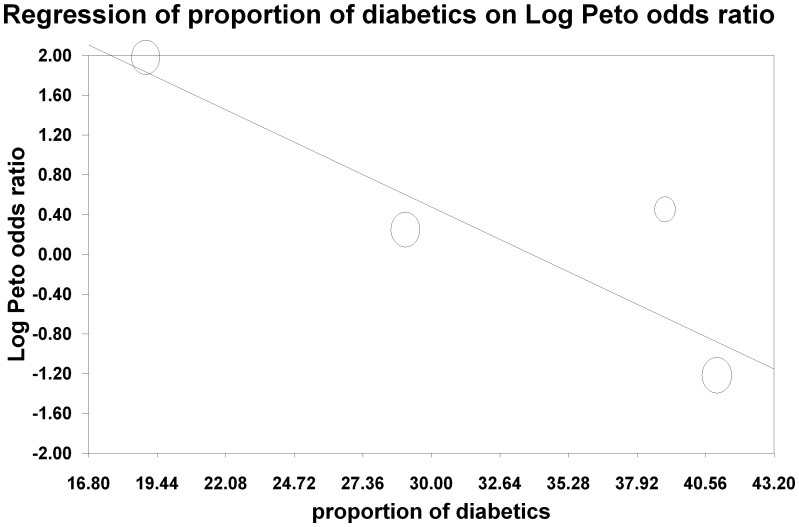
Metaregression of the proportion of patients with diabetes in a trial on the X-axis against the log Peto odds ratio on the Y-axis. This shows that there is a significant trend to greater benefit with oral volume expansion in trials with a higher proportion of diabetic patients.

The influence of each study was estimated by deleting each in turn from the analysis and noting the degree to which the pooled effect size changed. We considered a study influential if its exclusion changed our conclusion or the effect estimate by at least 20% [24], [36]. Two studies [18], [32] did change the effect size by the prespecified 20%, however neither reached statistical significance. Excluding the study by Cho et al [18], which had a well defined protocol for oral sodium formulations and water as part of the oral volume expansion protocol, resulted in a shift of the effect size towards benefit with the intravenous arm (OR 1.70, 95% CI 0.71, 4.07, p = 0.23). Exclusion of the Trivedi et al [32] study, which did not have a prespecified oral volume expansion protocol, resulted in a shift in the other direction, towards a benefit with the oral route (RR 0.75, 95% CI 0.37, 1.50, p = 0.42). Metaregression based on prespecified covariates (diabetes, baseline kidney function, contrast dose, average age) was not significant for baseline kidney function (p = 0.71) or age (p = 0.1). There was a significant trend with contrast dose (p = 0.01) (Figure 5) which suggested a beneficial effect of intravenous over oral volume expansion in trials where high contrast volume was used. There was also a significant trend with diabetes, with a beneficial effect of oral over intravenous volume expansion in trials with a higher proportion of diabetic patients (p<0.05) (Figure 6).

### Renal Replacement Therapy, Prolonged Hospitalization and Mortality

Four trials (n = 386 patients) [18], [29], [32], [33] reported the need for acute dialysis following contrast exposure. None of the patients in either arm of these trials needed acute dialysis during the hospital stay. Two trials (n = 144 patients) reported data on hospitalization. Trivedi [32] et al reported that one patient in the IV arm required prolonged hospitalization versus three in the oral arm. Cho [18] et al reported length of stay which was not significantly different (4.1 days with IV versus 5.6 days with oral). Only one study reported in-hospital mortality; there were no deaths in the 91 patients in that study [18].

## Discussion

We found no significant difference between pre-procedural intravenous and oral volume expansion for prevention of CI-AKI. The observed heterogeneity was resolved if we included only studies with a prespecified standard protocol for oral volume expansion. However, these findings are limited by the small sample size and the paucity of endpoints in the included trials. The metaregression also suggests a possible interaction between high contrast volume and protective effect of intravenous volume expansion.

The oral route of volume expansion has been used successfully in other clinical settings, such as correction and prevention of hypovolemia from gastrointestinal losses as well as with physical activity in hot and dry environments [37], [38], [39]. It also has considerable advantages over the intravenous route. It can easily be prescribed for outpatient procedures and it does not require nursing and day-care resources [19], [22]. It would also be more likely to be implemented compared to intravenous volume expansion due to this logistical ease [18]. Lastly, it would likely result in a net saving in terms of health care resources. On the other hand, compliance with oral volume expansion would be harder to measure, especially in a real world setting outside clinical research.

The advantage of the oral route is magnified when radiocontrast procedures are performed in the out of hospital setting. Indeed, CT scanners are often located in free-standing centers – less than 50% of the CT scanners are located at hospitals in a major metropolitan city in India [21]. Indeed, some cardiac catheterization laboratories are also located in free-standing facilities in the United States [40].

Our results also suggest that a pre-defined protocol for oral volume expansion may be more effective than just general advice to allow unrestricted fluid intake. Using sodium along with water also may be more effective than using water alone in CI-AKI prevention. Two studies have reported the effect of oral sodium on urinary indices. One of the studies, included in this analysis, collected 24-hour urinary sodium excretion and reported comparable urinary sodium excretion with oral salt compared to intravenous saline [29]. Another study has compared urinary alkalinization with oral compared to intravenous bicarbonate and found it to be similar [41]. These data suggest that the changes in urinary physiology might be similar regardless of the route of sodium and water administration, thus imparting a similar protective effect.

Patients at greater risk of an adverse outcome also exhibit greater benefit with an effective intervention [42] and we tested this by performing metaregression for selected covariates but with mixed results. Baseline kidney function is a robust predictor of CI-AKI events [7], [8], [10], [11], [12], [43] but we did not find any association between the benefit with either the intravenous or oral route and baseline kidney function. The volume of contrast administered has been identified in previous observational studies as a predictor of CI-AKI events, with doses greater than 100 ml [6] or exceeding a safe dose threshold (based on weight and serum creatinine [44]) being associated with AKI [45]. We did find a significant trend in favor of intravenous volume expansion in patients receiving a higher dose of contrast, suggesting intravenous volume expansion may be more beneficial in studies where patients receive large amounts of contrast. Lastly, diabetes also is an established risk factor for CI-AKI and has been included in risk scores for CI-AKI [46]. Intriguingly, we found a benefit with oral volume expansion in studies with a higher proportion of diabetes. This result may have been biased because of one study with a low proportion of diabetics who received a high contrast dose [32]. Additionally, this is a study level effect and may be an ecologic fallacy that could be clarified with individual patient level data [47].

Data on clinically significant outcomes, such as long-term kidney function, need for renal replacement therapy, hospitalization and mortality was quite sparse in these studies. None of the patients in the four trials that reported this outcome needed renal replacement therapy, so it is very difficult to comment on the comparative effectiveness of the two strategies on this important outcome.

There are important limitations to this meta-analysis. Our literature search only revealed a small number of eligible trials and the total number of CI-AKI events was only 45 in the 513 patients studied. None of the individual trials had a sample size greater than 290 or more than 20 events, which have been criteria used for sample size calculation for trials in the CI-AKI literature [36], [48]. Additionally, the meta-analysis results changed significantly with exclusion of two individual studies, and in opposite directions, suggesting that the overall results are not quite robust. There were clinical differences amongst the volume expansion protocols used which resulted in statistical heterogeneity in the pooled results. However, this heterogeneity resolved in the stratified analysis. Lastly, the trial populations overwhelmingly involved patients undergoing cardiac catheterization, which makes these results difficult to generalize to patients at lower risk of AKI, such as those receiving intravenous contrast for a CT scan [49].

In conclusion, the oral route of volume expansion, especially when combined with sodium and administered in a specific protocol, may be as effective as the intravenous route. Due to the limitations of the included trials, however, the relative effectiveness of oral volume expansion versus intravenous volume expansion for the prevention of CI-AKI cannot be firmly stated. Given the potential advantages of oral (e.g. reduced patient burden and cost) over intravenous volume expansion, adequately powered trials comparing these strategies with clinically important outcomes need to be conducted.

## Supporting Information

Table S1 **Search strategy for MEDLINE search using the OVID search engine.**
(DOC)

Table S2 **PRISMA Checklist.**
(DOC)
