# Supplementary material for: Prevention of Contrast-Induced Acute Kidney Injury: Is Simple Oral Hydration Similar To Intravenous? A Systematic Review of the Evidence
Source: PLoS One. 2013 Mar 26;8(3):e60009. doi: 10.1371/journal.pone.0060009 (PMC3608617; doi:10.1371/journal.pone.0060009)
Supplement: Table S1 — Search strategy for MEDLINE search using the OVID search engine. (DOC) [file pone.0060009.s001.doc]

**Database: Ovid MEDLINE(R) In-Process & Other Non-Indexed Citations and Ovid MEDLINE(R) <1950 to Present> Search Strategy:**

--------------------------------------------------------------------------------

1 exp Contrast Media/

2 (contrast media or contrast medium or contrast material$ or contrast agent$ or contrast dye or radiographic contrast).tw.

3 (radiocontrast media or radiocontrast medium or radiocontrast agent$).tw.

4 or/1-3 (88075)

5 (nephritis or nephropath$ or nephrotoxic$).tw

6 ((impair$ or damag$ or reduc$ or injur$) adj2 (renal or kidney)).tw.

7 exp nephritis/ or diabetic nephropathies/

8 exp renal insufficiency/

9 Kidney Diseases/ci [Chemically Induced]

10 or/5-9

11 4 and 10

12 (contrast-induced nephr$ or contrast-associated nephr$).tw.

13 (Contrast-induced acute kidney injury or ci-aki).tw.

14 12 or 13

15 11 or 14

16 Infusions, Intravenous/ or Injections, Intravenous/ (

17 (intravenous or iv).tw.

18 exp Tomography, X-Ray Computed/

19 (comput$ adj3 tomograph$).tw.

20 (ct or cat).tw.

21 urography/ or (urography or pyelography).tw.

22 or/16-21

23 15 and 22

24 Epidemiologic studies/

25 exp case control studies/ or exp cohort studies/

26 case reports.pt.

27 Case control.tw.

28 (cohort adj (study or studies)).tw.

29 Cohort analy$.tw.

30 (Follow up adj (study or studies)).tw.

31 (observational adj (study or studies)).tw.

32 (Longitudinal or retrospective or cross sectional).tw.

33 Cross-sectional studies/

34 randomized controlled trial.pt.

35 controlled clinical trial.pt.

36 randomized.ab.

37 placebo.ab.

38 clinical trials as topic.sh.

39 randomly.ab.

40 trial.ti.)

41 review.pt.

42 meta-analysis.mp,pt.

43 (meta analys$ or metaanalys$).tw.

44 (systematic adj1 (review$ or overview)).tw.

45 cochrane database of systematic reviews.jn.

46 search$.tw.

47 or/24-46

48 23 and 47

49 animals/ not humans/

50 48 not 49
